# HIF‐1α promotes the proliferation and migration of pulmonary arterial smooth muscle cells via activation of Cx43

**DOI:** 10.1111/jcmm.17003

**Published:** 2021-10-26

**Authors:** Xiao‐Jian Han, Wei‐Fang Zhang, Qin Wang, Min Li, Chun‐Bo Zhang, Zhang‐Jian Yang, Ren‐Jie Tan, Li‐Jun Gan, Le‐Ling Zhang, Xue‐Mei Lan, Fang‐Lin Zhang, Tao Hong, Li‐Ping Jiang

**Affiliations:** ^1^ Key Laboratory of Drug Targets and Drug Screening of Jiangxi Province Nanchang China; ^2^ Institute of Geriatrics Jiangxi Provincial People’s Hospital Affiliated to Nanchang University Nanchang China; ^3^ Department of Neurology Jiangxi Provincial People’s Hospital Affiliated to Nanchang University Nanchang China; ^4^ Department of Pharmacy The Second Affiliated Hospital of Nanchang University Nanchang China; ^5^ Department of Pharmacology, School of Pharmaceutical Science Nanchang University Nanchang China; ^6^ Department of Neurosurgery First Affiliated Hospital of Nanchang University Nanchang China

**Keywords:** connexin 43, hypoxia, hypoxia‐induced pulmonary hypertension, migration, proliferation, pulmonary artery smooth muscle cells

## Abstract

The proliferation of pulmonary artery smooth muscle cells (PASMCs) is an important cause of pulmonary vascular remodelling in hypoxia‐induced pulmonary hypertension (HPH). However, its underlying mechanism has not been well elucidated. Connexin 43 (Cx43) plays crucial roles in vascular smooth muscle cell proliferation in various cardiovascular diseases. Here, the male Sprague‐Dawley (SD) rats were exposed to hypoxia (10% O_2_) for 21 days to induce rat HPH model. PASMCs were treated with CoCl_2_ (200 µM) for 24 h to establish the HPH cell model. It was found that hypoxia up‐regulated the expression of Cx43 and phosphorylation of Cx43 at Ser 368 in rat pulmonary arteries and PASMCs, and stimulated the proliferation and migration of PASMCs. HIF‐1α inhibitor echinomycin attenuated the CoCl_2_‐induced Cx43 expression and phosphorylation of Cx43 at Ser 368 in PASMCs. The interaction between HIF‐1α and Cx43 promotor was also identified using chromatin immunoprecipitation assay. Moreover, Cx43 specific blocker (^37,43^Gap27) or knockdown of Cx43 efficiently alleviated the proliferation and migration of PASMCs under chemically induced hypoxia. Therefore, the results above suggest that HIF‐1α, as an upstream regulator, promotes the expression of Cx43, and the HIF‐1α/Cx43 axis regulates the proliferation and migration of PASMCs in HPH.

## 1 INTRODUCTION

Chronic hypoxia‐induced pulmonary hypertension (HPH), an incurable disease, is often a complication in patients with chronic heart failure, chronic obstructive pulmonary disease and sleep apnoea.^1^ Pulmonary vascular remodelling plays an important role in HPH pathology, which is mainly characterized by thickening of the pulmonary artery wall and stenosis of the lumen.^2^ Proliferation and migration of pulmonary artery smooth muscle cells (PASMCs) is the main cause of pulmonary vascular remodelling.^3^ The inhibition of PASMCs proliferation and migration is expected to be the main pathway for PH treatment.

Connexins (Cxs) are transmembrane proteins that oligomerize to form a pore in the cell membrane known as a hemi‐channel and interact with small regulatory molecules such as 3',5'‐cyclic adenosine monophosphate (cAMP), adenosine 5' triphosphate (ATP), calcium (Ca^2+^) and inositol 1,4,5‐triphosphate (IP3), which can pass directly through the cell membrane. In the vascular system, the core Cxs are Cx37, Cx40 and Cx43, and deletion of these genes causes severe cardiovascular abnormalities.^4^ Cx43 has been shown to engulf vascular smooth muscle cells (VSMCs), resulting in proliferation phenotypes and the development of atherosclerosis.^5^ Recent studies have demonstrated that intimal up‐regulation of Cx43 induces VSMCs proliferation in atherosclerotic plaques through gap junction generation.^6^ In cardiovascular and cerebrovascular diseases, Cx43 up‐regulation and smooth muscle coupling has been observed, thereby inducing proliferation and migration through up‐regulation of intercellular communication.^7^ These findings collectively suggest that alterations in the regulation of Cx43 expression could underlie VSMCs dysfunction.

The role of Cx43 in pulmonary hypertension (PH) has attracted increasing attention.^8^ A previous study investigated the role of endothelial Cx43 in pulmonary vascular reactivity in PH, focussing on pulmonary endothelial function and the interaction of pulmonary artery endothelial cells (PAECs) and PASMCs.^9^, ^10^ Unfortunately, the biological role of Cx43 in VSMCs function of PH remains largely unknown. In the present study, we established the hypoxia model by culturing PASMCs with cobalt chloride (CoCl_2_) or by exposing SD rats to 10% O_2_ and then observed changes in proliferation and migration under hypoxia. In addition, the effects of Cx43 on CoCl_2_‐treatment PASMCs dysfunction, as well as the underlying mechanism, were also explored.

## 2 MATERIALS AND METHODS

### 2.1 *Rat* *model of HPH*

After one week under normoxia, SD rats were randomly and equally divided into two groups which named hypoxia group and control group (n = 8 in both groups). Rats were exposed to continuity hypoxia (FIO_2_ of 0.10) in an isobaric hypoxic chamber for up to 21 days in hypoxia group while maintained in a normal oxygen condition (FIO_2_ of 0.21) for the same time in control group, according to the same methods used in our previous studies.^11^ After established HPH rat model, the right ventricle (RV) was separated from left ventricle and septum (LV + S) and weighed. The extent of RVH was calculated as the ratio of RV to (LV + S). In all rats, the freshly isolated pulmonary arterial samples were frozen in liquid nitrogen for mRNA and protein expression analysis. The right lower lung which used in haematoxylin‐eosin (HE) staining and in situ hybridization was fixed in 4% paraformaldehyde.

### 2.2 *Histological* *and Immunohistochemistry analysis*

HE staining of right lung was detected in accordance to the same method used in our previous study.^11^ In brief, the fixed lungs were sliced mid‐sagittally and embedded in paraffin and then cut into approximately 5 μm thick sections by microtome. Then, sections were placed on glass slides and stained by haematoxylin and eosin for morphometric analysis and visualization under an Olympus BX41 microscope (Tokyo, Japan).

### 2.3 *Cell* *experiments*

Normal rat PASMCs were acquired from the American Type Culture Collection (ATCC, Mannassas, VA, USA). We subcultured the cells and used cells at the 4th to 9th passages for our experiments. The cells were cultured at 37℃ under 5% CO_2_ in high‐glucose Dulbecco's modified Eagle's medium (DMEM) containing 10% foetal bovine serum (FBS). Confluent cells were starved with serum‐free medium for 24 h. The control group consisted of cells cultured with no special treatment for the next 24 h, whereas the hypoxia group was cultured in serum‐free medium with 200 µM CoCl_2_.

Lentiviruses encoding Cx43‐shRNA (LV‐Cx43‐shRNA) or a negative control (LV‐NC‐shRNA) were constructed using the AdMax lentivirus system (Shanghai Genechem Co., Ltd., China) according to the manufacturer's protocol. PASMCs were infected with lentivirus for 72 h. The three target sequences of Cx43 shRNA are presented in Table 1. Viral titres were routinely concentrated to 2E+9 (TU/mL), as determined by enzyme‐linked immunosorbent assays (ELISAs). The Cx43 inhibition or hypoxia‐inducible factor (HIF)‐1α inhibition groups were treated with Cx43 inhibitor (^37,43^Gap27) or HIF‐1α inhibitor (echinomycin), respectively, for 1 h prior to the induction of hypoxia.

**TABLE 1:** Target sequences of Cx43 shRNA

Name	Sequence
Cx43 shRNA‐1	5'‐AGGAAGAGAAGCTAAACAA‐3’
Cx43 shRNA‐2	5'‐GCTGGTTACTGGTGACAGA‐3’
Cx43 shRNA‐3	5'‐AGAGCACGGCAAGGTGAAA‐3’

### 2.4 *Cell* *proliferation assay*

Cell proliferation was detected using the Titer 96R AQueous One Solution cell proliferation assay kit (Promega, USA). All protocols were performed according to the manufacturers’ instructions as described previously.^11^, ^12^ Three repeat holes were implemented in each group, and all results from three independent experiments.

### 2.5 *Wound*‐*healing* *assay*

The wound‐healing assay was carried out as described previously.^8^, ^13^ PASMCs were plated at a density of 8×10^4^ cells/well in 6‐well plates. After the cells reached 85% confluence, wounds were induced by scratching with 200‐µl pipette tips, followed by washing with the medium to remove cellular debris. Cells were then incubated in normoxia or hypoxia conditions. Wound closure was monitored by comparing digital photographs of the same region of interest taken at 0‐ and 24‐h time points. Pictures were analysed using the Image Pro Plus program. Cell migration was expressed as the percentage of wound area re‐covered after 24 h.

### 2.6 *Transwell* *migration assay*

PASMCs (5 × 10^4^ cells) were seeded into the upper chamber of a 24‐well Transwell filter with 8‐µm pore size. The lower chamber was filled with media containing 1% FBS, and cells were allowed to migrate through the membrane to the underside for 24 h and then fixed with 4% paraformaldehyde for 20 min. The non‐migrating cells on the upper side of the filter were removed with cotton swabs, and the cells that migrated through the pores of the filter were stained with 1% crystal violet for 30 min. Pictures of six different image fields per well were taken at 200× magnification to count the number of stained cells.

### 2.7 *Chromatin* *immunoprecipitation*

Normoxic or hypoxic (200 μM CoCl_2_ for 6 h) PASMCs were harvested and processed with the Simple ChIP Enzymatic Chromatin IP Kit (Cell Signaling Technology) according to the manufacturer's instructions. For HIF‐1α‐specific chromatin immunoprecipitation (ChIP), chromatin was immunoprecipitated with 5 µg mAb (BD Transduction Laboratories). We used histone H3‐like protein (RPL30) as a positive control and IgG as a negative control (NC). The relative amounts of chromatin immunoprecipitated by the anti‐HIF‐1 Abs were determined by reverse transcription PCR and SYBR Green quantitative PCR (qPCR) using specific primers for the Cx43 gene promoter. Primer sequences are presented in Table 2.

**TABLE 2:** Primer sequences for PCR

Primer names	Forward primer	Reverse primer
Cx43	TCTGCCTTTCGCTGTAACACT	GGGCACAGACACGAATATGAT
HIF‐1α	ACCCTCTGATTTAGCATGTAG	GTAGGTTTCTGCTGCCTTGT
β‐actin	AGTCCCTCACCCTCCCAAAAG	AAGCAATGCTGTCACCTTCCC
Cx43 promoter	AACCGACGAGTAGACATACC	TGACCTAATTCTCCTCCTCT
RPL30	GGCACTGTTGATGGTCTATGG	TGCGTAAGGACTGCTGATACT
LgG	ACCACTGTGCAAATCTTTC	ACACCACACATATAGAACCT

### 2.8 *SYBR* *Green quantitative PCR analysis*

Total RNA was extracted by TRIzol (Invitrogen, Carlsbad, CA), and 500 ng RNA was subjected to reverse transcription using the Prime Script reverse transcription reagent kit (DRR037A; TaKaRa) according to the manufacturer's instructions. Quantitative analysis of the change in expression levels was performed using SYBR Premix Ex Taq (DRR041A; TaKaRa) with the ABI Prism 7300 system (Applied Biosystems). PCR cycling conditions were an initial incubation at 95°C for 30 s, followed by 40 cycles of denaturation at 95°C for 5 s and annealing at 60°C for 31 s. Data analysis was performed by the comparative Ct method using the 7300 system SDS software. β‐actin was used as an endogenous control. The primers used for qRT‐PCR are presented in Table 2.

### 2.9 *Western* *blot analysis*

Proteins were extracted from cultured PASMCs with RIPA buffer (containing 1% PMSF) for 30 min on ice, and equal amounts of protein were separated by 10% SDS‐polyacrylamide gels and transferred onto PVDF membranes. Western blots were performed using primary antibodies for Cx43 (Abcam, 1:500), phosphorylation of Cx43 at Ser 368 (Affinity, 1:500), HIF‐1α (Beijing ZSBio, 1:500) or β‐actin (Millipore, 1:1000) and the horseradish peroxidase (HRP)‐coupled goat anti‐mouse secondary antibody (for Cx43, Promega, 1:2000) or HRP‐coupled goat anti‐rabbit secondary antibody (for HIF‐1α and β‐actin, Beijing ZSBio, 1:5000). The chemiluminescence signals were detected with the Luminata™ Crescendo substrate (Millipore, WBLUR0100). Densitometric analysis was conducted with a ChemiDoc XRS+System (Bio‐Rad Co. Ltd. USA).

### 2.10 *Statistical* *analysis*

Data are presented as the mean ± SEM. Statistical analysis was performed by Student's t test for two groups or ANOVA followed by the Student‐Newman‐Keuls test for multiple groups. *p *< 0.05 was considered statistically significant. All statistical analyses were performed using SPSS19.0. All experiments were performed at least three independent times.

## 3 RESULTS

### 3.1 *Cx43* *and phosphorylation of* *Cx43* *at Ser 368 (p*‐*Cx43) were up*‐*regulated in HPH rat model*

**FIGURE 1:**
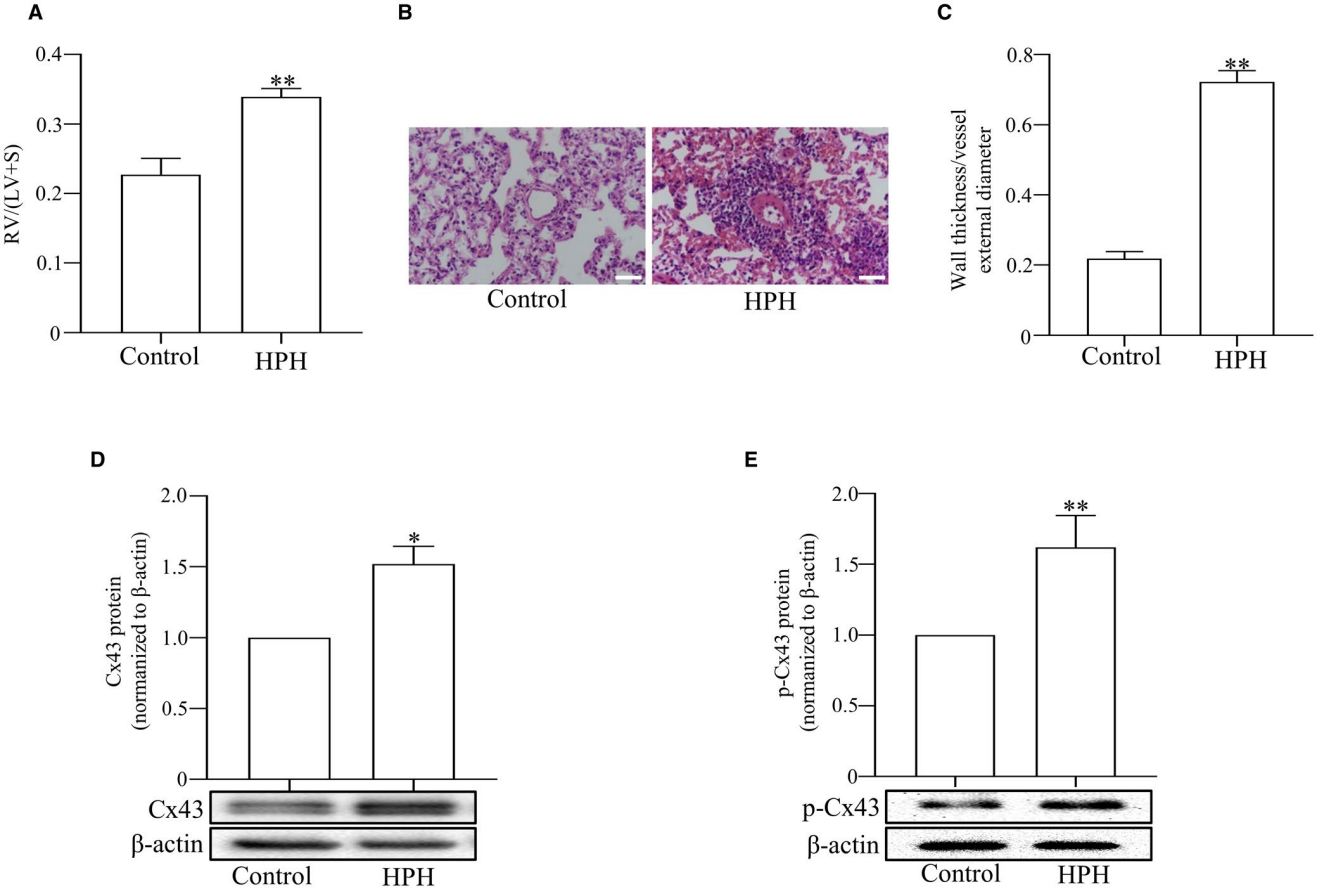
The expression of Cx43 and p‐Cx43 in HPH rat model. Hypertrophy of right ventricle (RV) was calculated by the ratio of RV weight to that of left ventricle (LV) plus interventricular septum (S) in each group (A). Small intrapulmonary arteries remodelling measured by HE staining in lung sections of rats (B). The ratio of wall thickness to total vessel external diameter in the lung sections from each group (C). The levels of Cx43 and p‐Cx43 in the pulmonary artery of rats were measured by Western blot (D and E). Scale bar, 50 μm. Data are means ± SEM, ^*^ *p *< 0.05, ^**^ *p *< 0.01 vs. Control

As same as our previous studies, HPH rat model was successfully obtained after 3 weeks exposure to hypoxia presented with hypertrophy of right ventricle (Figure 1A) and small intrapulmonary arteries remodelling (Figure 1B,C). In our established HPH models, we found the expression of Cx43 and p‐Cx43 in pulmonary artery was significantly up‐regulated than in a normoxic rats (Figure 1D,E).

### 3.2 *Hypoxia* *up*‐*regulated Cx43 and phosphorylation of Cx43 at Ser 368 expression in PASMCs*

**FIGURE 2:**
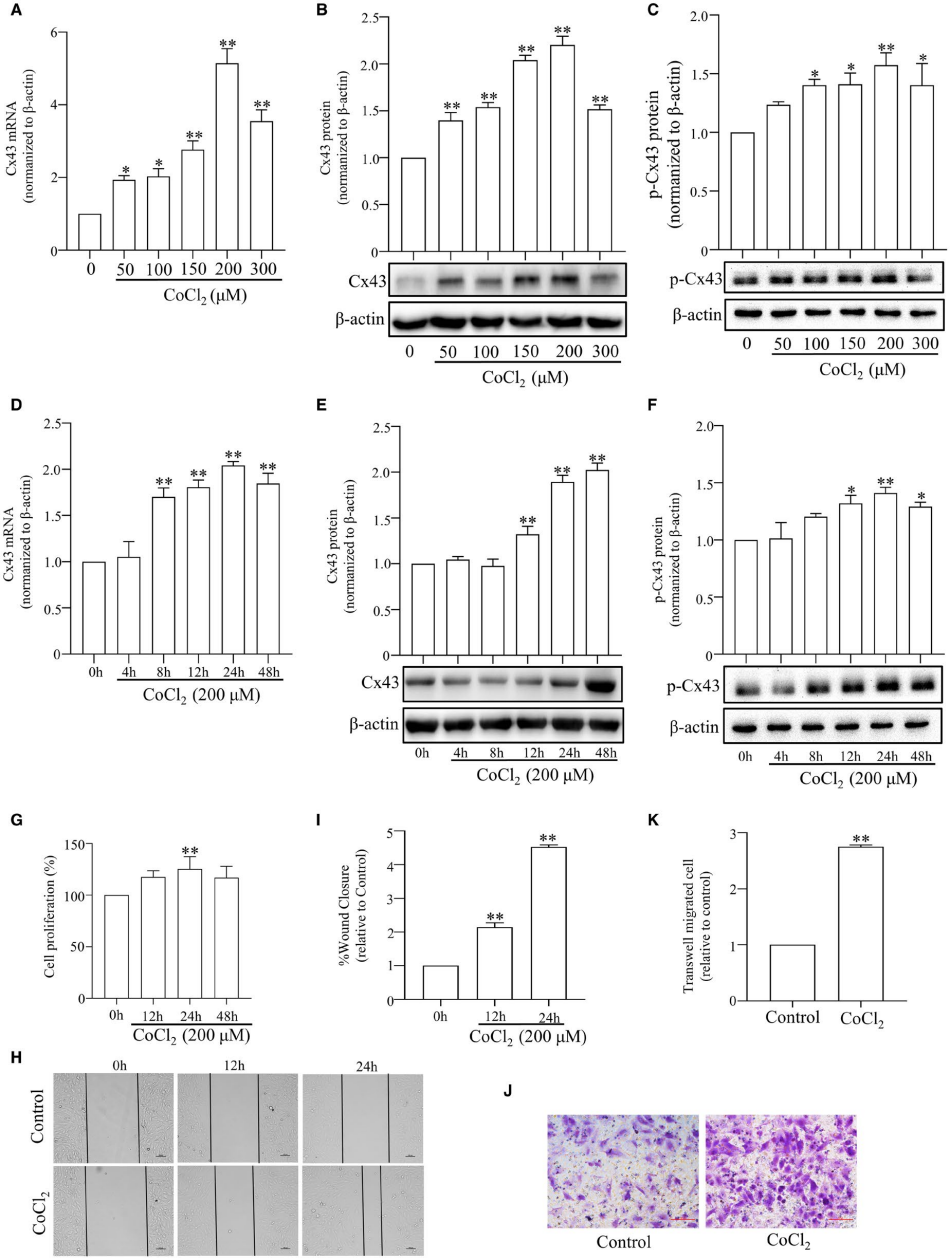
Effect of various concentrations of CoCl_2_ and treatment times on the expression of Cx43 and p‐Cx43, the proliferation and migration of PASMCs. The levels of Cx43 mRNA, Cx43 protein and p‐Cx43 in the PASMCs after CoCl_2_ stimulation at various concentrations (0, 50, 100, 150, 200 and 300 μM) were measured by qRT‐PCR (A) and Western blot (B and C). The levels of Cx43 mRNA, Cx43 protein and p‐Cx43 in the PASMCs after 200 μM CoCl_2_ stimulation for 0, 4, 8, 12, 24 and 48 h were measured by qRT‐PCR (D) and Western blot (E and F). Proliferation of PASMCs was measured by MTS assay (G). Migration of PASMCs was measured by Wound‐healing assay (H and I) and Transwell migration assay (J and K). All results from three independent experiments. Scale bar, 100 μm. Data are means ± SEM, ^*^ *p *< 0.05, ^**^ *p *< 0.01 vs. Control

Hypoxia has been reported to regulate Cx43 expression and channel activity in different cell models.^14^, ^15^ However, no data are currently available addressing the role of Cx43 channels in PASMCs in the context of a hypoxic microenvironment and their putative involvement in cell proliferation and migration. Here, we analysed the effect of hypoxia on the expression of Cx43 and p‐Cx43 in PASMCs. First, we examined the concentration‐dependent effect of CoCl_2_ (50, 100, 150, 200 and 300 μmol/L) for 24 h. As illustrated, CoCl_2_ at a dose of 50 μmol/L significantly up‐regulated the expression of Cx43 mRNA and protein, and the maximum effect was reached at 200 μmol/L (Figure 2A,B). Similarly, we found that CoCl_2_ at a dose of 100 μmol/L significantly up‐regulated the level of phosphorylation of Cx43 at Ser 368, and the maximum effect was also reached at 200 μmol/L (Figure 2C). According to the results, PASMCs were treated with 200 μmol/L CoCl_2_ for subsequent experiments. To investigate the time‐dependent effect, we exposed PASMCs to 200 μmol/L CoCl_2_ for 0, 4, 8, 12, 24 and 48 h. Hypoxia up‐regulated the levels of Cx43 mRNA and protein at 8 h (Figure 2D) and 12 h (Figure 2E) respectively. The level of phosphorylation of Cx43 at Ser 368 was also up‐regulated at 12 h (Figure 2F). Furthermore, hypoxia also induced PASMCs proliferation (Figure 2G) and migration (Figure 2H–K) in a time‐dependent manner. These data indicate that Cx43 may play a role in PASMCs proliferation and migration.

### 3.3 *Hypoxia* *induced PASMC proliferation and migration through Cx43*

**FIGURE 3:**
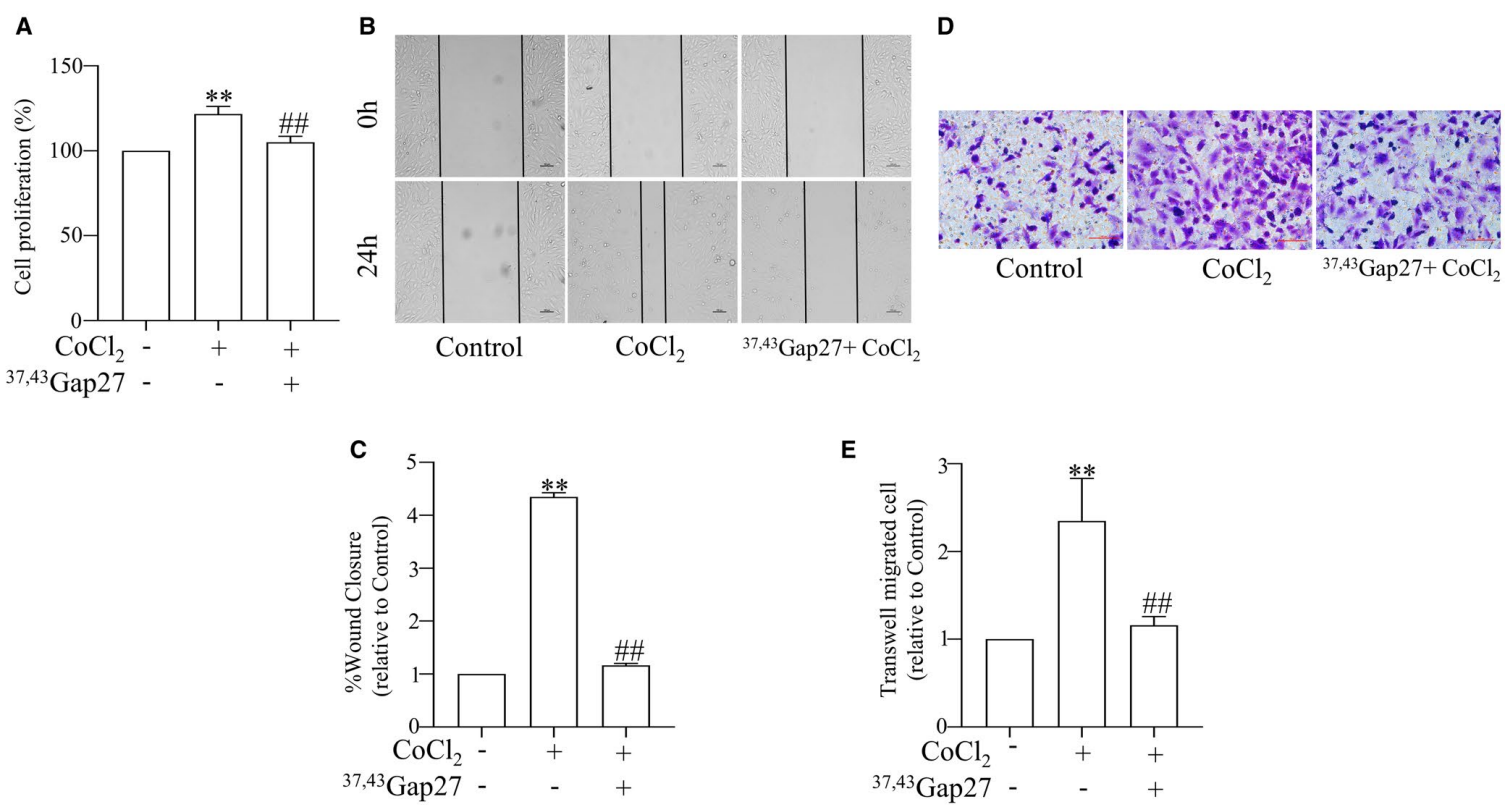
^37,43^Gap27 prevented CoCl_2_‐treatment PASMCs proliferation and migration. Proliferation of PASMCs was measured by MTS assay (A). Migration of PASMCs was measured by Wound‐healing assay (B and C) and Transwell migration assay (D and E). All results from three independent experiments. Scale bar, 100 μm. Data are means ± SEM, ^**^ *p *< 0.01 vs. Control, ^##^ *p *< 0.01 vs. CoCl_2_

To determine the role of Cx43 in PASMCs proliferation and migration, we manipulated Cx43 by knocking down its expression with Cx43‐targeting shRNA or ^37,43^Gap27, which is a specific Cx‐mimetic peptide blocker of Cx37 and Cx43. As shown in Figure 3A, exposure to hypoxia for 24 h remarkably induced the proliferation of PASMCs, which was inhibited by ^37,43^Gap27. Wound‐healing and Transwell assays showed that ^37,43^Gap27 prevented PASMCs migration by CoCl_2_‐treatment (Figure 3B–E).

**FIGURE 4:**
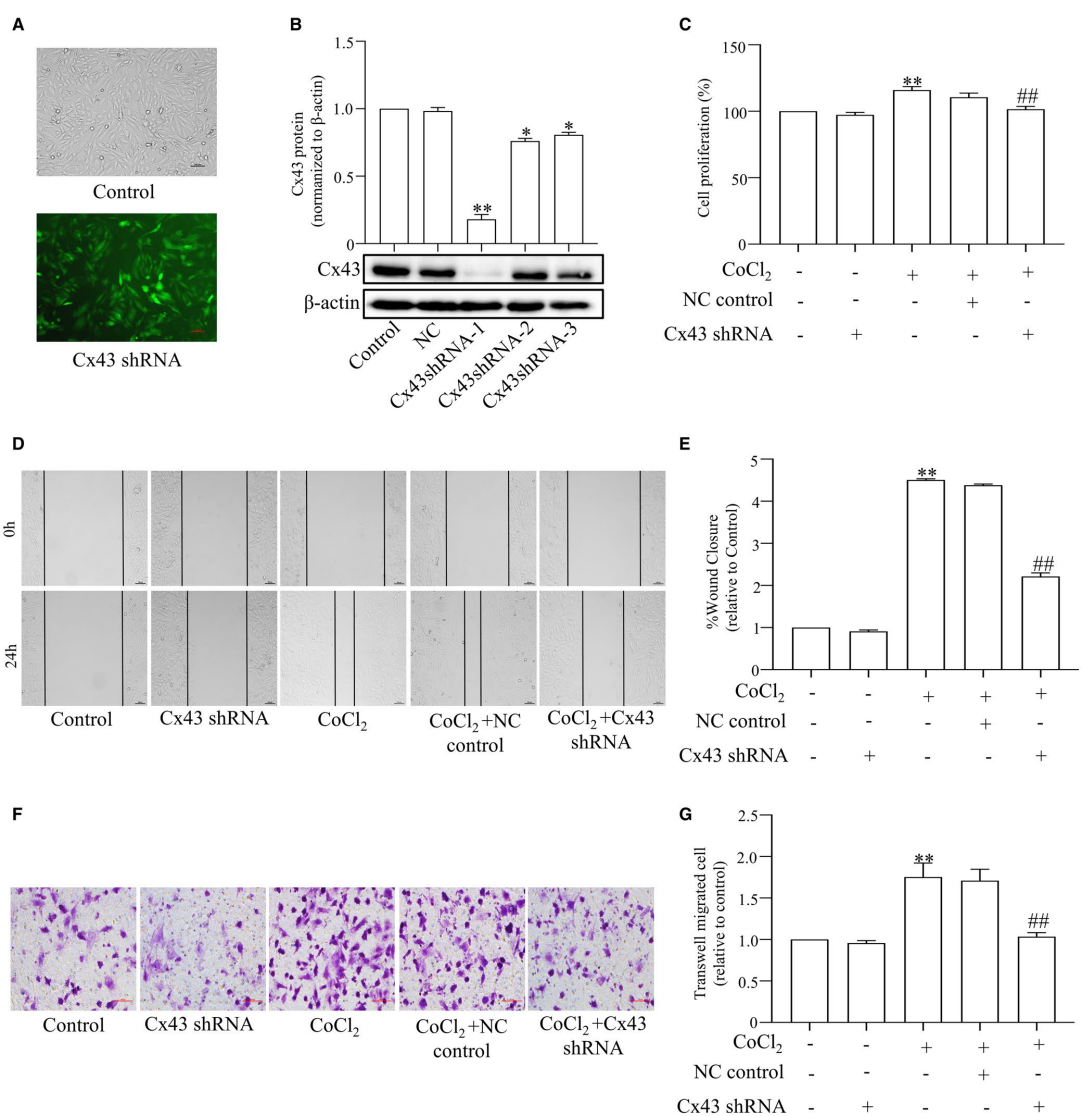
Cx43 shRNA prevented CoCl_2_‐treatment PASMCs proliferation and migration. The infection of Cx43 shRNA‐1 encoded in lentivirus in PASMCs and Cx43 expression level in cells was determined by fluorescence microscope (Scale bar, 100 μm) and Western blot respectively (A and B). Proliferation of PASMCs was measured by MTS assay (C). Migration of PASMCs was measured by Wound‐healing assay (D and E) and Transwell migration assay (F and G). All results from three independent experiments. Scale bar, 100 μm. Data are means ± SEM, ^**^ *p *< 0.01 vs. Control, ^##^ *p *< 0.01 vs. CoCl_2_

Then, we used the Cx43 shRNA to specifically block the Cx43 gene. Figure 4A shows that transfection was successful. We transfected different fragments into PASMCs and found that fragment 1 of shRNAs significantly decreased the protein expression of Cx43 (Figure 4B). Therefore, we chose to use fragment 1 of shRNAs for subsequent experiments. The genetic block of Cx43 by specific shRNA produced a comparable effect to ^37,43^Gap27. As shown, exposure to hypoxia significantly induced PASMCs proliferation (Figure 4C) and migration (Figure 4D–G), which were reversed by transfection of Cx43 shRNA. Taken together, these results suggest that Cx43 up‐regulation contributes to PASMCs proliferation and migration.

### 3.4 *Hypoxia* *increased Cx43 promoter activity through the HIF*‐*1α transcription factor*

**FIGURE 5:**
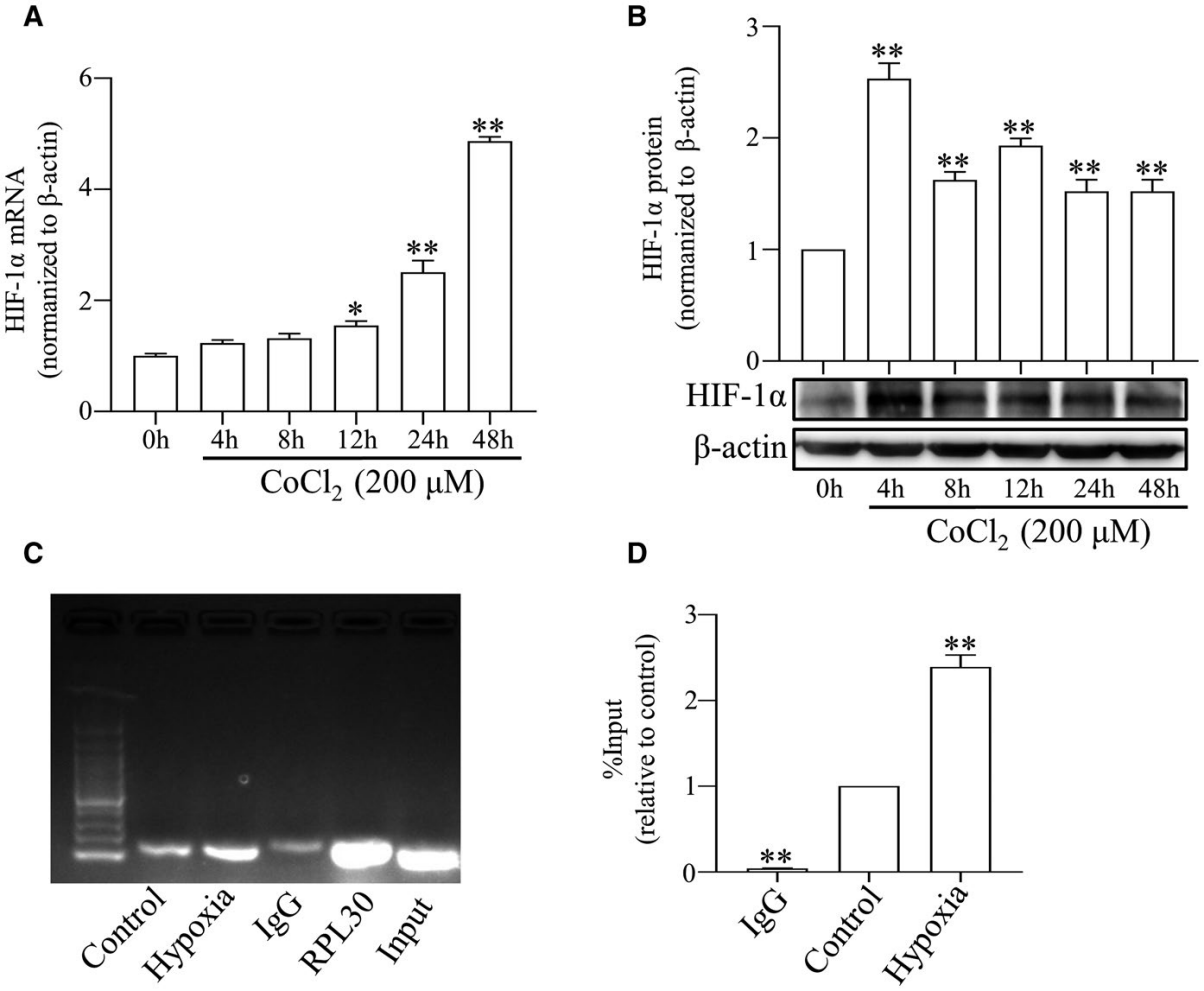
Hypoxia facilitates the interaction between HIF‐1α transcription factor and Cx43 promoter. The expression of HIF‐1α in the PASMCs after 200 μM CoCl_2_ stimulation for 0, 4, 8, 12, 24 and 48 h was measured by qPCR (A) and Western blot (B). HIF‐1α CHIP/input DNA ratios for Cx43 promotor were measured by RT‐PCR (C) and qPCR (D). All results from three independent experiments. Data are means ± SEM, ^*^ *p *< 0.05, ^**^ *p *< 0.01 vs. Control

To pinpoint the molecular mechanism by which hypoxia up‐regulates the expression of Cx43, we next focussed on HIF‐1α, which is a hallmark of HPH and promotes the progress of vascular remodelling through regulating PASMCs proliferation and migration.^16^ We first tested whether hypoxia regulates HIF‐1α expression in PASMCs. As shown, hypoxia significantly increased HIF‐1α mRNA (Figure 5A) and protein expression (Figure 5B), and these changes were correlated with the induction of Cx43. Since HIF‐1α can bind to the Cx43 promoter region, we performed ChIP analysis to show that hypoxic conditions induced more binding of HIF‐1α protein to the Cx43 promoter compared with control conditions (Figure 5C,D). Therefore, our data indicate that HIF‐1α is involved in the induction of Cx43 by hypoxia.

### 3.5 *Hypoxia* *increased the expression of Cx43 in PASMCs via HIF*‐*1α*‐*dependent transcriptional activation*

**FIGURE 6:**
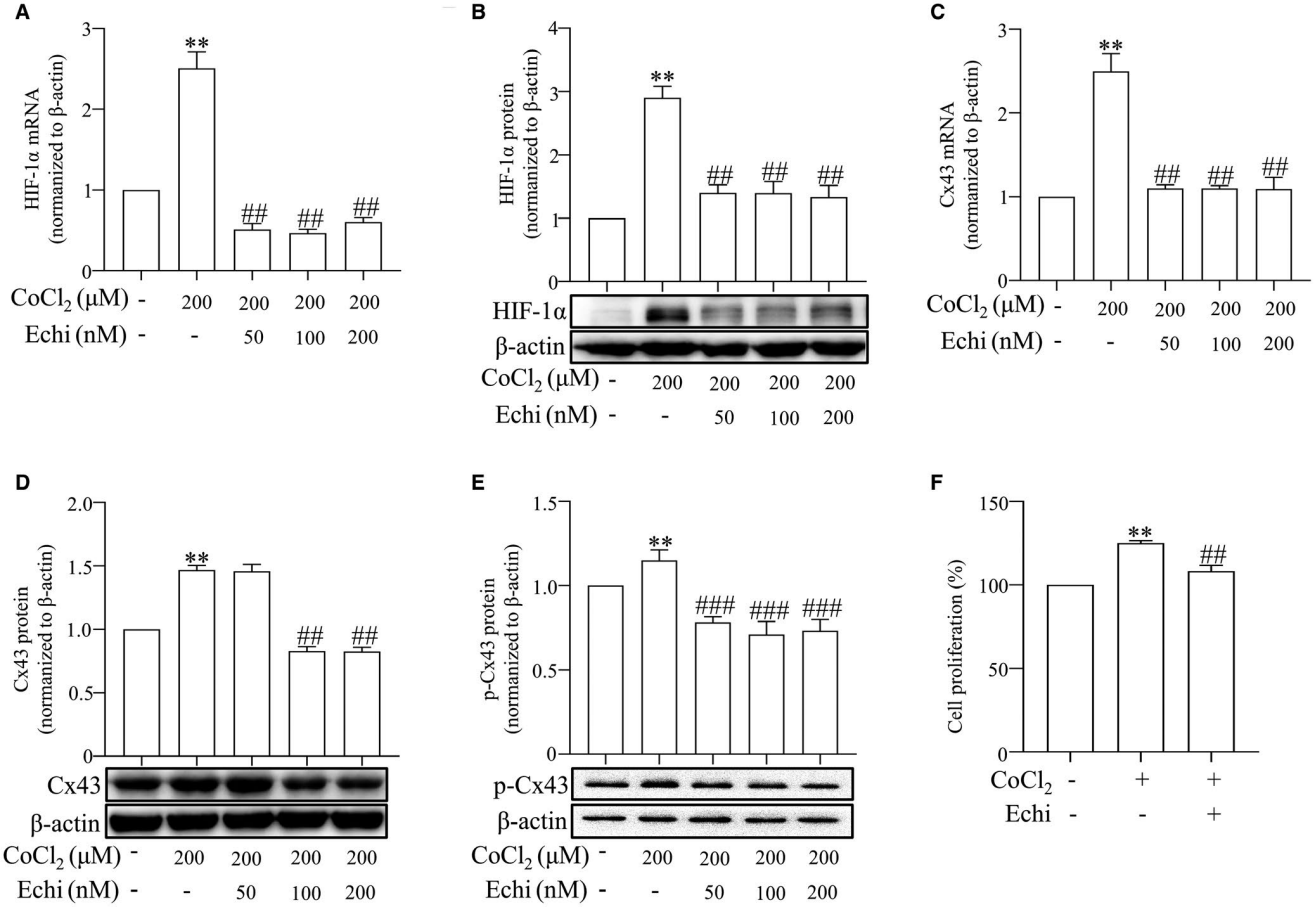
Echinomycin prevented CoCl_2_‐treatment Cx43 and p‐Cx43 expression and PASMCs proliferation. The expression of HIF‐1α in the PASMCs after CoCl_2_ (200 μM) and echinomycin at various concentrations (0, 50, 100 and 200 μM) was measured by qPCR (A) and Western blot (B). The levels of Cx43 mRNA, Cx43 protein and p‐Cx43 in the PASMCs after CoCl_2_ (200 μM) and echinomycin at various concentrations (0, 50, 100 and 200 μM) was measured by qPCR (C) and Western blot (D and E). Proliferation of PASMCs was measured by MTS assay (F). All results from three independent experiments. Data are means ± SEM, ^**^ *p *< 0.01 vs. Control, ^##^ *p *< 0.01, ^###^ *p *< 0.001 vs. CoCl_2_

**FIGURE 7:**
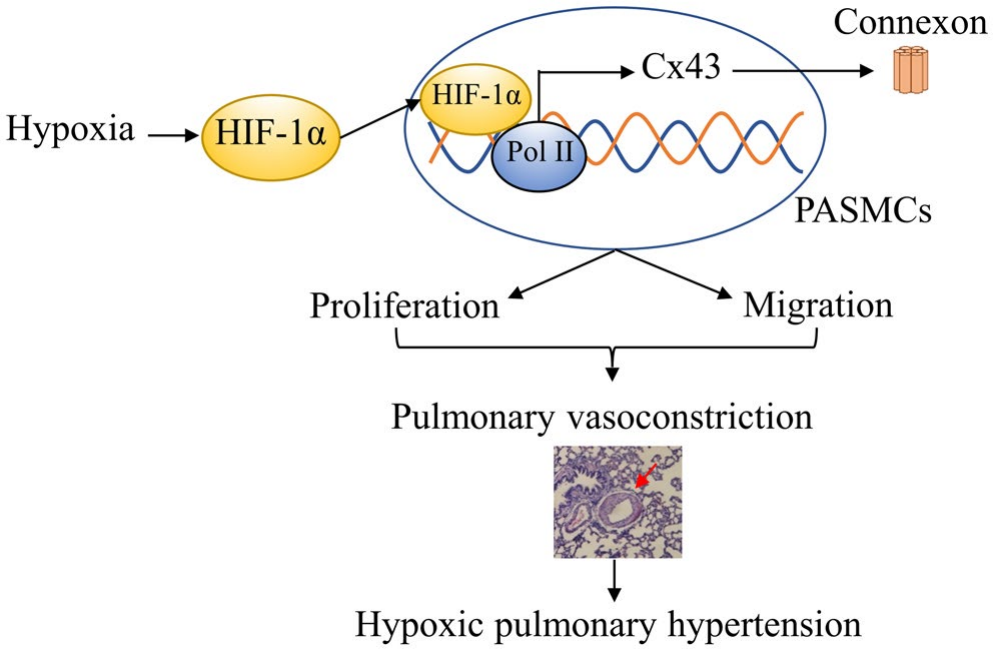
Scheme illustrating the role of HIF‐1α/Cx43 in hypoxia‐induced the proliferation and migration of PASMCs

Previous studies have shown that elevated pressure contributed to the increase of Cx43 protein expression in cultured cells from aorta and Cx43 expression in the lung was increased in Sprague‐Dawley rats exposed to chronic hypoxia (CH) or treated with monocrotaline (MCT).^17^, ^18^, ^19^, ^20^ Therefore, to further identify the effect of HIF‐1α on the regulation of Cx43, we examined the effects of different concentrations of the HIF‐1α inhibitor echinomycin (50–200 nmol/L) on PASMCs (Figure 6A,B) and found that echinomycin eliminated the hypoxia‐promoting effect on Cx43 and p‐Cx43 (Figure 6C–E). Furthermore, echinomycin also inhibited CoCl_2_‐treatment PASMCs proliferation (Figure 6F), showing a similar effect as Cx43 inhibition. Taken together, these results imply that hypoxia up‐regulates Cx43 in a HIF‐1α‐dependent manner and then promotes the proliferation and migration of PASMCs, which subsequently leads to hypoxic pulmonary vasoconstriction and pulmonary hypertension (Figure 7).

## 4 DISCUSSION

To our knowledge, this is the first study to show that Cx43 plays an important role in hypoxia‐induced PASMCs proliferation and migration in PH. Several reports have suggested a prominent role for Cx43‐mediated VSMCs function, such as proliferation, migration, phenotypic switch and contractile reactivity, in various diseases such as atherosclerosis^21^ and hypertension. Previously, the study showed that Cx43 played a key role in vascular endothelial cell function. For example, asymmetric dimethylarginine induces pulmonary endothelial dysfunction via changes in Cx43 expression and activity.^22^ In addition, Cx43 is involved in nitric oxide‐induced pulmonary vascular relaxation and plays a gender‐specific and agonist‐specific role in pulmonary vascular contractility. Taken together, these findings add to the growing body of evidence that suggests that Cx43 is involved in the regulation of the pulmonary vasculature.

Emerging evidence indicates that both genetic and environmental factors are major factors for PH development and progression. Prolonged exposure to alveolar hypoxia, at a high altitude or secondary to chronic pulmonary diseases, is a potent environmental stimulus for patients with PH.^23^ Vasoconstriction induced by acute hypoxia leads to a reversible increase in pulmonary vascular resistance, and prolonged hypoxia triggers the proliferation and migration of PASMCs, subsequently facilitating vascular remodelling and persistent vasoconstriction.^24^ To explore the mechanisms responsible for the development of HPH and to effectively treat this condition, clinical observations and animal experiments are commonly performed. In previous studies using cell models, injury caused by chemically induced hypoxia was used to construct HPH models.^25^, ^26^ CoCl_2_ is a well‐known hypoxia mimetic agent that mimics the hypoxic response in many aspects.^25^, ^26^ In the present study, we examined the effects of CoCl_2_ at various concentrations and treatment durations on the number of viable PASMCs, and we selected a concentration of 200 μM for a treatment duration of 24 h to establish the cell model of hypoxia, as was done in a previous study.^27^

PH and cancers share similar phenotypes; both are characterized by increased cell proliferation and migration. In this study, we chose Cx43 as the research focus because previous studies have indicated its pro‐tumour function in different types of cancers.^28^ Consistently, up‐regulation of Cx43 and phosphorylation of Cx43 at Ser 368 were observed in hypoxia‐treated PASMCs in the present study. Moreover, blocking the function of Cx43, ^37, 43^Gap27 or shRNA resulted in a significant suppression of PASMCs proliferation and migration under hypoxic conditions. Our findings support the hypothesis that Cx43 offers a pro‐proliferation effect by up‐regulating the level of Cx43 protein expression and phosphorylation of Cx43 at Ser368.

In the current study, we found that hypoxia up‐regulated Cx43 gene, the level of Cx43 protein expression and phosphorylation of Cx43 at Ser368, followed by proliferation and migration in PASMCs. In contrast, in cardiomyocytes, hypoxia decreased Cx43 and then induced cell death.^29^ In many cancer cells, hypoxia can downregulate the originally highly expressed Cx43, thereby inhibiting proliferation and migration and promoting apoptosis and autophagy.^28^, ^30^ In addition, studies have been reported that Cx43 expression was increased in rat pulmonary artery fibroblasts (rPAFs) and pulmonary arteries (PAs) from chronic hypoxia‐induced pulmonary hypertension patients, but decreased in PAs from idiopathic pulmonary arterial hypertension (IPAH) patients and rat pulmonary artery smooth muscle cells (rPASMCs).^20^, ^31^ Interestingly, however, Tanshinone IIA promotes PASMCs apoptosis via up‐regulating Cx43 levels during hypoxia and reverses vascular remodelling.^32^ Although the effect of hypoxia on the expression of Cx43 differs, the pro‐proliferation and anti‐apoptosis effects of Cx43 are consistent across various cell types.

Hypoxia‐inducible factor‐1α, which is highly regulated by the intracellular O_2_ concentration, plays a critical role in regulating PASMCs phenotypes under hypoxia exposure.^23^ In response to hypoxia, the transcription factor HIF‐1α is rapidly stabilized and translocated to the nucleus, where it binds to the hypoxia‐responsive element (HRE) motif (5‐RCGTG‐3) to induce the transcription of many critical genes to sustain cell survival under hypoxic stress.^33^ A previous study verified many HIF‐1α target genes, such as GLUT‐1, GLUT‐3, Hx‐1, Hx‐2 and Cx43.^33^ Hypoxic stress reportedly increases the expression of Cx43 by transcriptional activation through HIF‐1α in melanoma cells in cancer.^30^ Here, we identified Cx43 as one of the targets of HIF‐1α in PH. HIF‐1α induces Cx43 expression and its phosphorylation at Ser 368 activity in PASMCs through binding to the promoter of the Cx43 gene (GJA1). We provide a novel physiological role for HIF‐1α in proliferation and migration of PASMCs through its induction of Cx43 expression and phosphorylation of Cx43 at Ser 368 activity. In line with this result, we showed that CoCl_2_‐treatment induced the expression of Cx43 in PASMCs in a time‐dependent manner. Furthermore, ^37, 43^Gap27 and Cx43 shRNA also efficiently alleviated the proliferation and migration of PASMCs via CoCl_2_‐treatment.

Research shows that HIF‐1α is involved in hypoxia‐induced PASMCs proliferation and migration.^34^, ^35^ Also, we observed an increase in HIF‐1α protein expression after 4 h of hypoxia, and then, its level declined slightly at 8–48 h but remained higher than in normoxia. However, mRNA level of HIF‐1α significantly up‐regulated at 12 h–48 h. We are surprised by the results of mRNA and protein level of HIF‐1α in PASMCs after hypoxia. It is possible that the transcription of HIF‐1α mRNA is slower than the ubiquitination degradation of HIF‐1α under hypoxia, which leads to the peak of HIF‐1α mRNA at 24–48 h and the peak of HIF‐1α protein at 4 h. c‐Src, a non‐receptor tyrosine kinase involved in the regulation of cell proliferation, has been reported to up‐regulate HIF‐1α expression. Interestingly, increased Cx43 expression inhibits c‐Src activity in rat glioma cells^36^ and astrocytes.^37^ Our results suggest that reduced HIF‐1α expression is accompanied by a decrease in the levels of Cx43 mRNA, Cx43 protein and phosphorylation of Cx43 at Ser368, showing the existence of a negative feedback regulation between HIF‐1α and Cx43 in PASMCs.

The present study demonstrates that hypoxia promotes the proliferation and migration of PASMCs via the HIF‐1α/Cx43 axis. Expression of Cx43 and its phosphorylation activity are necessary for PASMCs proliferation and migration under hypoxia. These findings suggest that Cx43 is a novel therapeutic target for PH.

## CONFLICT OF INTERESTS

The authors have declared that no competing interest exists.

## AUTHOR CONTRIBUTION

**Xiaojian han:** Funding acquisition (equal); Investigation (lead); Methodology (equal); Project administration (lead); Writing‐original draft (lead). **Weifang Zhang:** Investigation (supporting); Methodology (equal); Project administration (supporting); Writing‐original draft (equal). **Qin Wang:** Investigation (supporting); Methodology (equal); Project administration (supporting); Writing‐original draft (supporting). **Min Li:** Investigation (supporting); Methodology (equal); Project administration (supporting); Writing‐original draft (supporting). **Chunbo Zhang:** Investigation (supporting); Methodology (supporting); Visualization (equal). **Zhangjian Yang:** Investigation (supporting); Methodology (supporting); Visualization (equal). **Renjie Tan:** Methodology (supporting); Visualization (supporting). **Lijun Gan:** Methodology (supporting); Visualization (supporting). **Leling Zhang:** Methodology (supporting); Visualization (supporting). **Xuemei Lan:** Methodology (supporting); Visualization (supporting). **Fanglin Zhang:** Methodology (supporting); Visualization (supporting); Writing‐original draft (supporting). **Tao Hong:** Funding acquisition (equal); Project administration (equal); Writing‐review & editing (equal). **Liping Jiang:** Conceptualization (equal); Funding acquisition (equal); Writing‐review & editing (equal).

## Data Availability

The authors confirm that the data supporting the findings of this study are available within the article.
